# Intermittent Recovery of Severe Acute Aortic Regurgitation Arising From Infective Endocarditis

**DOI:** 10.7759/cureus.10462

**Published:** 2020-09-15

**Authors:** Christopher Cordeiro, Siddhant Trehan, Joseph N Heaton, Prema Bezwada, Samir Garyali

**Affiliations:** 1 Internal Medicine, The Brooklyn Hospital Center, Brooklyn, USA; 2 Cardiology, The Brooklyn Hospital Center, Brooklyn, USA

**Keywords:** infective endocarditis, aortic insufficiency

## Abstract

This case reports a 47-year-old male with a history of IV drug abuse, presenting with one week of left lower back pain. During the initial treatment, the patient became hemodynamically unstable, requiring vasopressor support. Transthoracic echocardiography (TTE) revealed a 1 cm x 1 cm aortic valve vegetation with severe aortic regurgitation and potential perforation of the valve leaflet. After hemodynamic stability was achieved, the patient left against medical advice, refusing urgent valvular surgery. Subsequent follow-up unveiled repeated recurrence of symptoms and surgical repair of the aortic valve.

## Introduction

Aortic regurgitation occurs due to abnormalities in the aortic leaflets, supporting structures in the aortic root and annulus, or both, leading to inadequate closure of the valve leaflets [1]. Common etiologies include infective endocarditis, rheumatic fever, congenital or senile valvular abnormalities, and aortic dissection. Typically, aortic regurgitation is more common in males and above 50 years of age [2]. When caused by infective endocarditis, affected individuals tend to be younger and have a history of IV drug abuse.

In acute aortic regurgitation (AAR), retrograde blood flow may overwhelm the heart's ability to pump, leading to cardiac output collapse and retrograde congestion [3]. Patients may present with signs mimicking acute heart failure, including dyspnea, palpitations, syncope, and chest pain. Additional signs may include a low pitched early diastolic murmur, absent S1, soft S2, audible S3, and water hammer pulses, though sensitivity and specificity for each may vary [4]. Echocardiography is the preferred diagnostic method for confirming AAR, with the additional benefit of providing insight into underlying etiology [5]. Hemodynamic support and emergent interventions may be warranted in severe acute presentations. When caused by endocarditis, treatment typically requires immediate valvular surgery.

## Case presentation

A 47-year-old male with a past medical history of IV heroin abuse, untreated hepatitis C, and Crohn's disease presented with a one-week history of left lower back pain. The pain was sharp, rated 5/10, nonradiating, without known relieving or exacerbating factors. Further history taking revealed five to six bags of IV heroin use per day for an unknown period, including on the morning of admission. Additional history and review of systems were noncontributory. Objectively, the patient was febrile at 102.6°F, had leukocytosis, prominent lactic acidosis, and an unremarkable chest X-ray. Empiric antibiotic therapy was initiated using vancomycin, ceftriaxone, and gentamicin. On day 2 of admission, the patient became hemodynamically unstable, refractory to IV fluid therapy, and received escalated care using vasopressor support.

**Figure 1:**
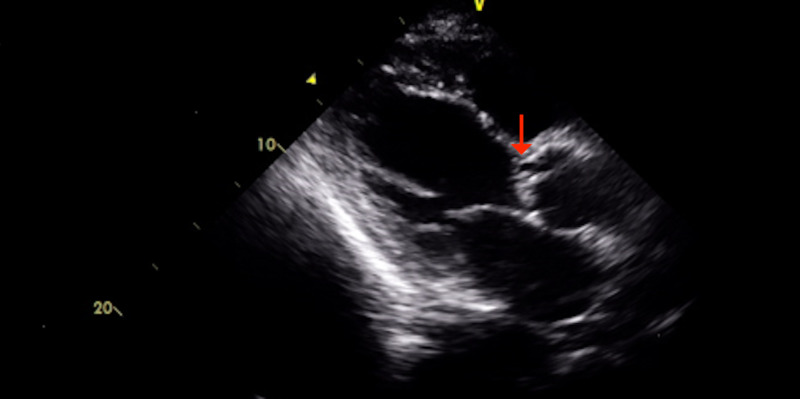
Parasternal long-axis view revealing the aortic valve vegetation and leaflet perforation.

**Figure 2:**
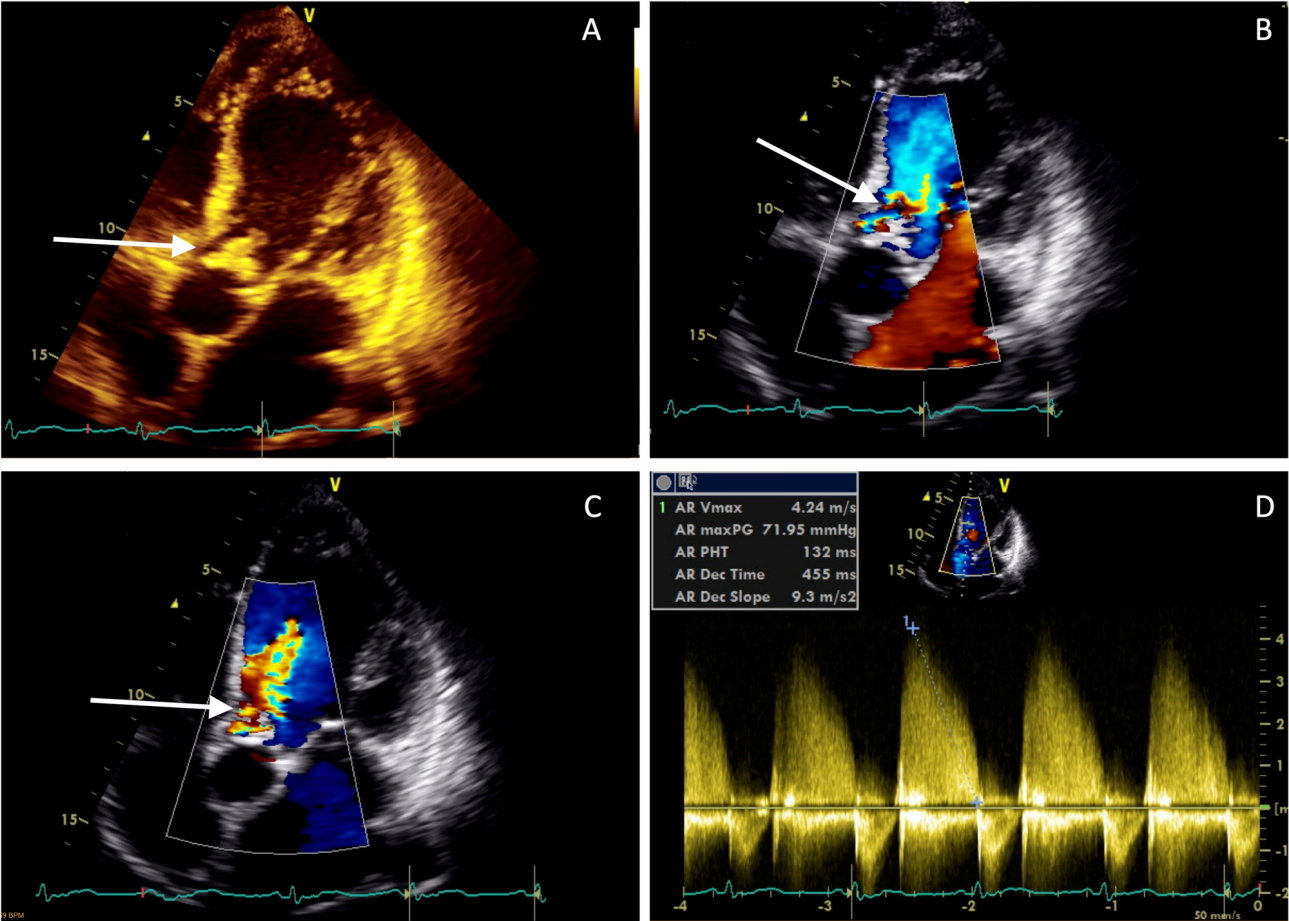
Aortic regurgitation using Doppler imaging. A: Apical five-chamber view displaying the valve perforation B, C: Severe aortic regurgitation using color Doppler flow imaging D: Measurement of aortic regurgitation using spectral Doppler flow

Blood cultures on admission grew *Streptococcus mitis*, sensitive to ceftriaxone; therefore, antibiotics were appropriately narrowed. The patient subsequently developed angina symptoms, where additional workup revealed troponin elevation to 0.82 without electrocardiogram changes, for which heparin was initiated. Transthoracic echocardiography (TTE) was remarkable only for aortic valve thickening, suspicious for vegetation, and moderate to severe aortic regurgitation. Transesophageal echocardiography was recommended for further investigation; however, after discussing the risks and benefits, the patient refused. Interval changes on a chest X-ray revealed new pulmonary edema. Repeat TTE showed an left ventricular ejection fraction (LVEF) of 60%-65% with a 1 cm x 1 cm aortic valve vegetation, severe aortic regurgitation, and valve leaflet perforation (Figures 1-2). The results, plan, and prognosis were discussed with the patient. After hemodynamic stabilization, the patient refused surgical intervention and left against medical advice with the prescribed antibiotic therapy. A month later, the patient was readmitted and reported that he received an aortic valve replacement two weeks after leaving the hospital. A subsequent TTE was performed and showed an LVEF of 55%-60% with a bioprosthetic aortic valve.

## Discussion

Acute aortic regurgitation develops from abnormalities in the valve leaflets or the aortic root [1]. The mechanism of AAR secondary to endocarditis is through valvular destruction, perforation, and abscess formation. A recent study found up to 50% of patients develop cusp perforation, flail, or both [6-7]. The improper valvular function instigates an abrupt increase in left ventricular volume, decrease in cardiac output, hypotension, and cardiogenic shock [8].

A thorough history of presenting illness may reveal chest or back pain complaints, cough, dyspnea, and palpitations, with symptoms mimicking heart failure. The physical exam can exhibit hypotension with widened pulse pressure, a decrescendo diastolic murmur, and a soft S2. Less commonly, physical exam findings may include an Austin-Flint murmur, bounding peripheral pulses, and head bobbing [4].

Electrocardiogram findings may be generic, with only nonspecific ST-T wave changes; ST deviations may be present due to a concurrent myocardial infarction or induced demand ischemia [9].

Echocardiography provides a definitive assessment of aortic regurgitation and can be done at the bedside, providing prompt evaluation. Echocardiography is optimally performed using parasternal long-axis and short-axis views, which offer an ideal survey of volume status and valvular structures, including the aortic valve, outflow tract, and aorta [5, 10]. The severity of AAR is determined by the extent of regurgitation from the left ventricle, effective regurgitant orifice area (ROA), and the regurgitant flow fraction. Doppler echocardiography affords 95% sensitivity and near 100% specificity in identifying regurgitant flow, using pulsed Doppler or continuous wave techniques [5,11]. In M-mode, early closure of the mitral valve indicates increased left ventricular end-diastolic pressure [10].

When evaluating suspected endocarditis etiologies, TTE is the best initial imaging choice, despite lower sensitivities for identifying valvular vegetations. Further imaging using transesophageal echocardiography is reasonable if there is high suspicion, and the transthoracic approach is indeterminate.

Unstable AAR requires urgent surgical intervention and is associated with lower mortality when accomplished promptly [12-15]. If a delay in surgical intervention is unavoidable, IV vasodilators and inotropic agents may provide a bridge to definitive therapy; beta-blockers and intra-aortic balloon pumps are contraindicated due to poor outcomes [16]. IV antibiotics should be initiated for treatment of endocarditis [17]. When considering valve replacement options, it is worthy to note that mechanical and prosthetic valve replacements have shown similar rates of success and reoccurrences of infection [15].

## Conclusions

Acute aortic regurgitation may be caused by a valvular pathology. Hemodynamically unstable cases require emergent surgical intervention to reduce overall mortality. In the presented case, the patient became hemodynamically unstable for a brief period and should have undergone the recommended procedure. The patient delayed treatment due to the intermittent absence of symptoms but ultimately required a bioprosthetic valve replacement. Consequently, this case shows the need for close monitoring and education about the importance of definitive treatment in severe AAR caused by infective endocarditis.
